# Supplementary figures and images for: A Comprehensive Analysis of Auxin Response Factor Gene Family in Melastoma dodecandrum Genome
Source: Int J Mol Sci. 2024 Jan 9;25(2):806. doi: 10.3390/ijms25020806 (PMC10815038; doi:10.3390/ijms25020806)

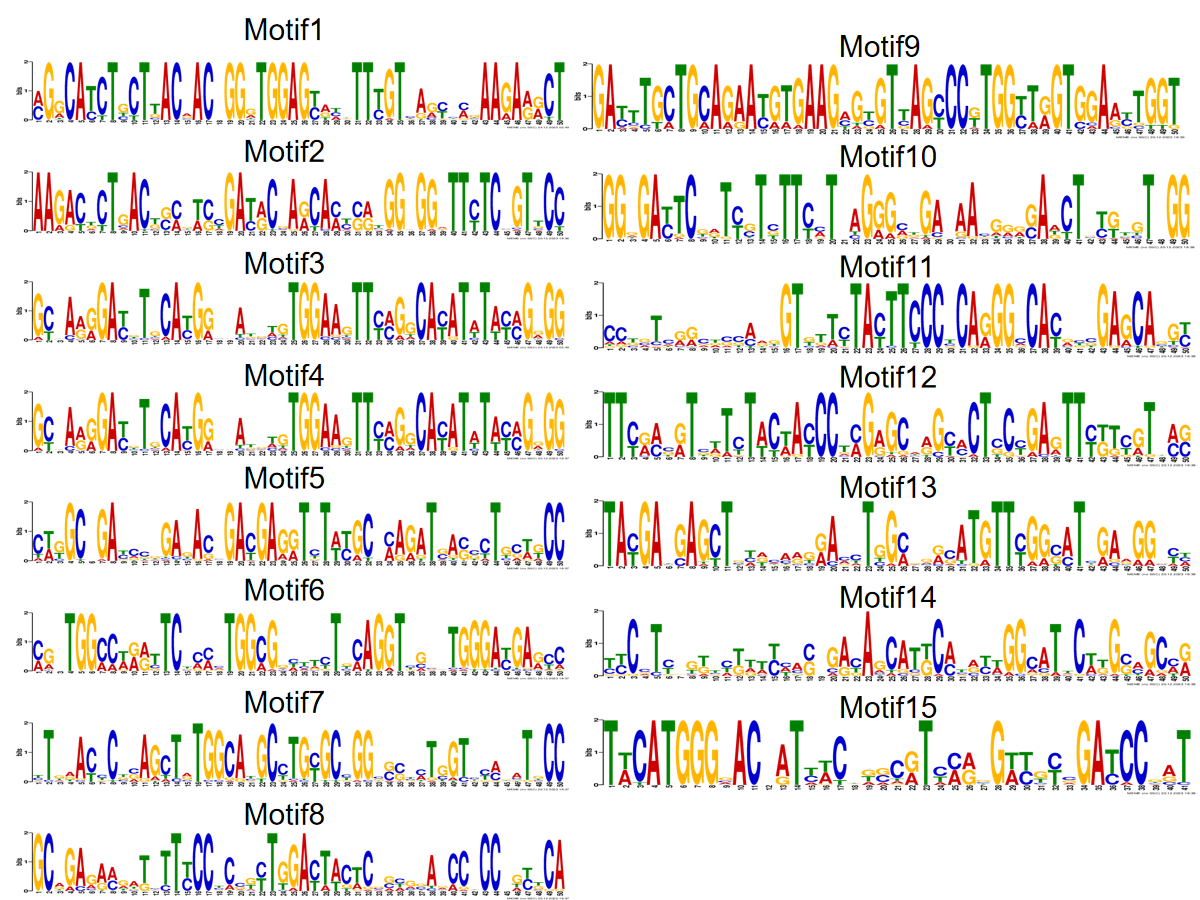

Supplement: Supplementary file 1 [file ijms-25-00806-s001.zip › Figure S2.tif]
